# Fruit Traits Reflect Adaptation to Dispersers Along a Tropical Elevational Gradient

**DOI:** 10.1002/ece3.72511

**Published:** 2025-12-02

**Authors:** Richard J. Hazell, Graham S. Kaina, Katerina Sam, Daniel Souto‐Vilarós, Wulan Koagouw, Vojtech Novotny, Mika R. Peck, Alan J. A. Stewart

**Affiliations:** ^1^ School of Life Sciences University of Sussex Falmer UK; ^2^ New Guinea Binatang Research Centre Nagada Harbour Madang Papua New Guinea; ^3^ Food and Agriculture Organization of the United Nations Project Office in Conservation and Environment Protection Authority Port Moresby Papua New Guinea; ^4^ Biology Centre of Czech Academy of Sciences Institute of Entomology Ceske Budejovice Czech Republic; ^5^ Faculty of Sciences University of South Bohemia Ceske Budejovice Czech Republic; ^6^ Science Research Initiative, College of Science University of Utah Salt Lake City Utah USA; ^7^ National Research and Innovation Agency (BRIN) KST B.J. Habibie Puspiptek Tangerang Indonesia

**Keywords:** dispersal syndromes, elevation, frugivory, fruit traits, gape limitation, phylogeny

## Abstract

Fruit traits have the potential to influence disperser communities and vice versa. Elevational gradients, where functional traits are known to change rapidly with distance, enable the study of these relationships at local scales. In this pilot study, we examine the trends in three fruit traits related to dispersal by frugivores: size, colour and presentation (i.e., location of displayed fruits on the trunk or on the branches) along a 200–2700 m asl rainforest elevation gradient in Papua New Guinea. We found fruit size to be lower at higher elevations. While specific fruit colours showed few significant elevational patterns, colours typically attributed to attracting avian dispersers were more prevalent at higher elevations. The proportion of ramiflorous species (bearing fruits from branches) increased with elevation. Finally, we use phylogenetic information to test the ‘dispersal syndromes’ hypothesis: that combinations of fruit traits have evolved in accordance with the preferences and sensory abilities of different frugivore guilds. All fruit traits except presentation showed little evidence of phylogenetic signal but we found fruits displaying colours attributed to attracting mammalian dispersers to be larger than ‘bird colour’ fruits. We found evidence for the correlated evolution of fruit size and colour, in support of the dispersal syndromes hypothesis. We encourage research at larger scales of time and space to further explore the relationships between fruit traits and frugivores, across elevational and other ecological gradients.

## Graphical abstract

This study investigates how fruit traits related to frugivore dispersal—specifically diameter, colour and presentation—vary along an elevation gradient in Papua New Guinea. Results showed that fruit diameter decreases with elevation, bird‐attracting colours are more common at higher elevations, and branch‐borne (ramiflorous) fruits increase with elevation. Phylogenetic analysis supports the ‘dispersal syndromes’ hypothesis, revealing a correlated evolution of fruit size and colour based on frugivore preferences, though most traits showed weak phylogenetic signals.

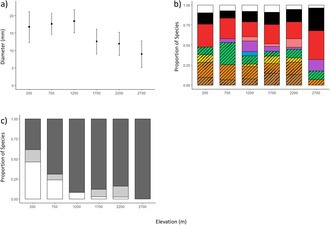

## 1 Introduction

The ability of a plant to disperse its seeds constitutes an important factor determining its survival (Janzen 1970; Connell 1971; Howe and Smallwood 1982; Beckman and Rogers 2013). In response, fruits have evolved into a variety of different forms in order to maximise seed dispersal ability in differing environments. In tropical regions, an estimated 70%–90% of plant species have evolved fleshy fruits that are adapted to dispersal by vertebrate frugivores (Muller Landau and Hardesty 2005). The frugivory mutualism thus has important implications for the evolution of fruit traits in tropical plants—traits related to accessibility and potential attractiveness to frugivores are of clear importance in determining a plant's fitness.

Tropical montane habitats may hold important clues into the relationships between fruit traits and dispersers. Elevational gradients in the tropics are characterised by steady but rapid changes in climatic conditions across relatively small geographical distances. This typically leads to high species turnover of both plant species and their potential dispersers and a corresponding high turnover of functional traits along tropical elevational gradients. Fruit trait profiles thus have the potential to influence, and, in turn, to be influenced by, local assemblages of frugivorous species occurring at different elevations (Dehling et al. 2014; Bender et al. 2018; Hazell et al. 2023; Korejs et al. 2025). Nevertheless, while changes in many plant functional traits across elevational gradients are well studied (Swenson and Enquist 2007; Hulshof et al. 2013; Asner et al. 2017; Homeier et al. 2021; Sigdel et al. 2023; Zhang et al. 2024), surprisingly little is currently known about how fruit traits change with elevation on a community level (but see Albert et al. 2018; Lu et al. 2019). Data on fruit traits are often less readily available than for other plant characteristics because fruits are physically difficult to reach, and present on plants only for short periods of time. If we are to fully understand the functional roles of plants in tropical forests, knowledge of fruit traits is a key component.

Of course, individual fruit traits do not exist in isolation. Numerous studies have attempted to detect the presence of ‘dispersal syndromes’. Akin to pollination syndromes (Fenster et al. 2004), the dispersal syndrome hypothesis states that combinations of fruit traits have evolved as a response to selective pressures from different frugivore guilds (Gautier‐Hion et al. 1985; Fischer and Chapman 1993; Lomáscolo et al. 2008, 2010; Valenta and Nevo 2020; Gómez‐Devia and Nevo 2024). For example, birds have acute colour vision and their gape size commonly limits the maximal size of fruits they can consume (Wheelwright 1985; Alcántara and Rey 2003). Mammals are typically larger than birds and can consume and disperse larger fruits. However, outside of the simian primates, mammals generally lack colour vision and rely more on olfactory cues to find fruits (Nevo et al. 2018). Finally, the smaller size of birds means they can easily feed on ‘ramiflorous’ fruits presented among the leaves at the ends of branches, while ‘cauliflorous’ fruits borne on the stem are more likely to be fed on by mammals such as bats (Whittaker and Jones 1994).

The island of New Guinea presents an ideal set of conditions for studying dispersal syndromes. The mountainous and relatively undisturbed terrain of its Central Range gives rise to fully forested elevational gradients exhibiting high ecological heterogeneity. Furthermore, it lacks primates, resulting in an exclusively nocturnal mammalian fauna, and creating a clear division of frugivores into a diurnal avian guild and a primarily nocturnal mammalian guild comprising bats, marsupials and rodents. If the matching of functional traits occurs here between fruits and their frugivore dispersers, we may expect to observe evidence of dispersal syndromes related to fruit colour and size. Bird‐dispersed fruits are hypothesised to be smaller and display colours that strongly contrast with a background of bark and foliage, aiding visual detection (Schmidt et al. 2004; Valenta and Nevo 2020). In contrast, as mammals are generally not gape‐limited, mammal‐dispersed fruits may be larger on average and display a greater range of sizes, reflecting the greater range of feeding techniques used by mammals (Howe 1986). Additionally, since visual cues are likely less important for mammals than for birds (Valenta et al. 2018), colour is expected to be more variable in mammal‐dispersed fruits and duller colours may occur.

Evidence for dispersal syndromes is mixed (e.g., Fischer and Chapman 1993; Lomáscolo et al. 2008, 2010; Valenta and Nevo 2020; Green et al. 2022; Rojas et al. 2022). An alternative non‐adaptive hypothesis is phylogenetic inertia, whereby fruit size and colour are determined by the size and colour of ancestral species, and frugivores disperse fruits according to pre‐determined preferences for certain combinations in a process known as ecological fitting (Janzen 1985; Jordano 1995; Flörchinger et al. 2010). If dispersal syndromes occur based on trait combinations selected for by dispersers, then we would expect evidence of correlated evolution of the traits in question, and the appearance of these traits independently in different clades. In contrast, the phylogenetic inertia hypothesis should predict phylogenetic clustering of these traits, indicative of shared evolutionary histories.

In this pilot study, we use a fruit trait dataset from a continuously forested elevational gradient in Papua New Guinea to address the following questions: (i) How do three key fruit traits related to dispersal by vertebrates (size, colour and presentation) change with elevation along the gradient? (ii) Can fruits along the elevational gradient be categorised into dispersal syndromes based on size and colour? (iii) To what extent are fruit traits along the gradient phylogenetically conserved? We hypothesise the following: (1) Fruit size will decrease with increasing elevation, as large dispersers, such as larger birds and frugivorous bats, are more abundant in the lowland areas than at higher elevations (Sam et al. 2019; Sivault et al. 2023). (2) Fruit colours associated with avian dispersal will be more prevalent at higher elevations, where birds are likely to play a dominant role in seed dispersal due to the relative scarcity of mammalian frugivores such as bats (Hazell et al. 2023; Sivault et al. 2023). (3) Because of the dominance of avian dispersers at higher elevations, ramiflorous fruits will similarly be more prevalent here, whereas cauliflorous fruits will be less frequent. (4) We will detect an association between fruit size and fruit colour categories corresponding to dispersal by either birds or mammals. (5) Fruit traits associated with dispersal by birds versus mammals will arise independently across the plant phylogeny, indicating convergent evolution of these traits.

## 2 Materials and Methods

### 2.1 Study Site

**FIGURE 1:**
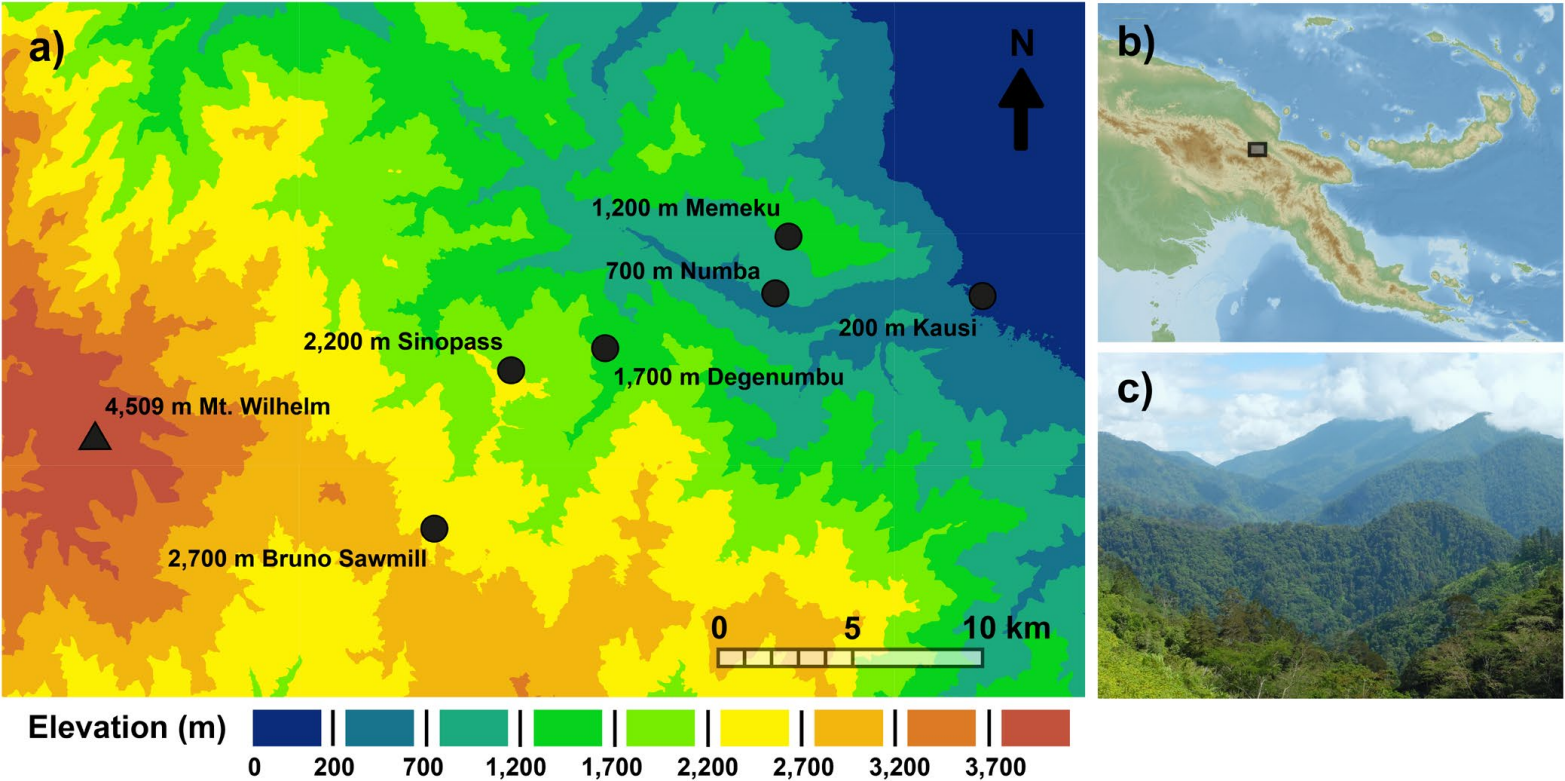
Study location. (a) Elevation map of the Mt. Wilhelm survey area, including named study sites, and coloured elevational bands in 500 m intervals as delimited by the elevations represented in our study. (b) Location of the survey area within Papua New Guinea (black rectangle). (c) Westward view along the elevational gradient in the direction of Mt. Wilhelm, depicting the continuous primary forest cover typical of the gradient. Elevation map created using ArcGIS Online using satellite data from Esri (2025). Relief map of Papua New Guinea by NordNordWest is licenced under CC‐BY‐SA‐3.0. Photo is by R. J. Hazell.

The study was conducted along the northeastern slopes of Mt. Wilhelm in the northern watershed of the Central Range of Papua New Guinea (Figure 1). The study area is a 20 km transect located in the Usino‐Bundi district of southern Madang province and comprises six study sites separated by 500 m elevation, ranging from 200 to 2700 m above sea level (asl) (5°44′ S, 145°20′ E; 5°49′ S, 145°09′ E). The sites represent the lower portion of a complete rainforest transect spanning from the lowland floodplains of the Ramu River to the treeline at 3700 m asl (Sam et al. 2019). The habitats at the surveyed sites range from lowland alluvial forest (200 m asl) through foothill forest (700–1200 m asl) and lower montane forest (1700–2200 m asl) to mid montane forest (2700 m asl) (Paijmans 1976). Mean annual temperature recorded across 3 years (2015–2017) using data loggers at each elevational site decreases from 24.9°C at 200 m to 14.3°C at 2700 m. Average annual precipitation measured by local weather stations is 3288 mm at 200 m asl, rising to 4400 mm at 3700 m asl, with a distinct condensation zone around 2500–2700 m asl (Sam and Koane 2014; Marki et al. 2016; Sam et al. 2019). Rainfall data for other elevations along the gradient were unavailable.

### 2.2 Fruit Surveys

We collected data on fruiting woody plants (trees, shrubs, lianas) and their fruits using transect surveys between March and July 2016. This time frame, corresponding to the first part of the dry season, is known from other parts of New Guinea to represent the period of peak fruiting (Wright 2005; Pangau‐Adam et al. 2021). Ten transects were created at each elevation, each 20 m wide and 500 m in length. This provided a total of 10 ha of sampled area per elevational site, and 60 ha across the gradient as a whole. Surveyors walked the transect route, searching carefully for any fruiting woody plants or fallen fruits. Fruiting plants were recorded only if the base of the stem occurred at least partially within the transect. When a fruiting plant was located, we collected data on plant location, diameter at breast height (DBH), growth form and taxonomy (identifying to species level where possible). No minimum DBH threshold was set. When necessary, leaf voucher specimens were collected and photographs of stems taken to allow subsequent detailed identification. We also collected information on the method of fruit presentation: either cauliflorous (fruiting directly from the stem) or ramiflorous (fruiting from the branches). In the case of fallen fruits encountered on the ground, we located the most likely source plant using binoculars. In most cases, this was not difficult as the plant was still displaying fruits. We collected up to 10 ripe fruits at random from across the canopy of each fruiting plant we encountered (mean = 3.9, SD = 2.8). In cases where fewer than 10 ripe fruits were reachable, we collected as many as safely possible. In cases where fruits were completely unreachable (*N* = 26 fruiting plants, 2.4% of total), we estimated mean fruit length and diameter and the colour of ripe fruits, using binoculars.

### 2.3 Fruit Measurement

We measured the collected fruits as soon as possible after collection to ensure that fruit traits were recorded prior to decomposition. Fruit dimensions were measured to the nearest 0.1 mm using digital callipers. Fruit diameter was chosen to represent fruit size in analyses. Diameter was defined as the secondary (longest orthogonal to the primary) axis, regardless of the fruit's morphological characteristics such as stem location or the orientation of seeds. Fruits were weighed using digital scales to the nearest 0.01 g. Fruit colour was defined subjectively using the basic colour categories of ‘red’, ‘orange’, ‘yellow’, ‘green’, ‘blue’, ‘purple’, ‘pink’, ‘brown’, ‘black’ and ‘white’. For bicoloured fruits, the dominant colour (covering > 50% of the fruit surface) was considered for analyses.

### 2.4 Analyses

To enable interspecific analyses of fruit traits across elevations, we first calculated the mean trait values per species at each elevation. For species occurring across multiple elevations, we calculated separate mean values for each elevation at which they were recorded, to allow for any potential intraspecific variation in fruit traits depending on elevation. In addition, we calculated fruit traits weighted by the number of individual fruiting plants at each elevation.

### 2.5 Analyses of Fruit Traits Across Elevations

We used generalised linear models (GLMs) to test for the effect of elevation on various fruit traits for both species and abundance‐weighted data. Analyses were conducted in R version 4.3.1 (R Core Team 2023). For fruit diameter, we used a GLM with a Gaussian error distribution and included plant DBH as a fixed effect. To test the effect of elevation on the proportion of fruiting plants bearing fruits of different colours and different presentation types, we ran separate GLMs for each colour and for each presentation type, using binomial error distributions. Additionally, we tested the effect of elevation on fruit colours when grouped into two categories (see ‘Fruit Syndromes’ below), again using a GLM with binomial error. Tukey pairwise comparisons were performed to adjust *p* values for multiple comparisons between elevations, calculated using the ‘emmeans’ function in the *emmeans* package (Lenth et al. 2018).

### 2.6 Phylogenetic Analyses

For phylogenetic analyses of fruit traits, we used a global phylogeny adapted by Smith and Brown (2018) from GenBank release 218 (ftp://ftp.ncbi.nlm.nih.gov/genbank) and the Open Tree of Life synthetic tree (taxonomy version 3; https://tree.opentreeoflife.org/about/synthesis‐release/v9.1). The phylogeny was subjected to hierarchical analysis with individual phylogenies constructed for major clades and using a backbone provided by Open Tree of Life version 9.1. We used the prune.sample function in the R package *picante* (Kembel et al. 2010) to subset this global phylogeny to include only species found in our dataset from the Mt. Wilhelm study sites.

In order to determine whether the categorical traits of colour and presentation were clustered or randomly distributed across the phylogeny, we used a null model analysis. We first calculated the mean phylogenetic distance (MPD) between individuals of each colour and of each presentation type and then compared this to a distribution of values generated by shuffling the tip labels across the phylogeny 999 times. We then assessed the deviation of observed and null values. Null models were implemented using functions in the package *picante*. For the continuous trait of fruit diameter, we used Pagel's lambda (λ) (Pagel 1999a) to test for phylogenetic signal in the trait data. Pagel's λ uses phylogenetic data to assess whether a trait has evolved independently of phylogeny (low phylogenetic signal) or if it conforms to an evolutionary model expected under Brownian motion (high phylogenetic signal) (Molina‐Venegas and Rodríguez 2017).

### 2.7 Fruit Syndromes

The fruit syndrome hypothesis predicts correlated evolutionary change in fruit size and colour according to dispersal guild (Lomáscolo et al. 2008). To test the hypothesis that fruit traits corresponding to dispersal by birds versus mammals evolved together, we first divided fruits into binary size and colour categories corresponding to each dispersal syndrome. Fruits were divided by colour into ‘mammal colour’ (brown, green, yellow and orange) and ‘bird colour’ (red, pink, purple, blue, black, white), according to Janson's (1983) classification. Fruits were divided by size based on mean fruit diameter for all fruiting plant species recorded in our data. We performed Pagel's likelihood ratio test of binary correlations (Pagel 1994, 1999b), using the subset of fruiting plant species for which phylogenetic data was available, on the two binary categories of colour (mammal vs. bird colour) and size (large vs. small), using the ‘fitPagel’ function in the package *phytools* (Revell 2012). Pagel's test compares the goodness of fit of a model of correlated evolution to one of independent evolution, considering phylogenetic branch lengths.

To test whether mammal colour fruits were larger overall than bird colour fruits (regardless of phylogeny), we performed a Mann–Whitney *U* test using fruit diameter per fruiting plant as the response variable. To test whether variation in fruit diameter was greater for mammal colour fruits than for bird colour fruits, we used a Fligner‐Killeen test, which is a non‐parametric test for assessing the homogeneity of variances (Fligner and Killeen 1976).

## 3 Results

We collected and measured ripe fruits from a total of 1062 fruiting plant individuals across all elevations, representing 167 plant species and morpho‐species. Twenty‐one species and morpho‐species were recorded from 200 m, 43 from 700 m, 38 from 1200 m, 33 from 1700 m, 38 from 2200 m and 28 from 2700 m asl (Table A1). Of these, 83 species were sufficiently identified to be used in phylogenetic analyses. Outside of phylogenetic analyses, we used data from all fruiting plant species and morpho‐species.

### 3.1 Fruit Size and Elevation

**FIGURE 2:**
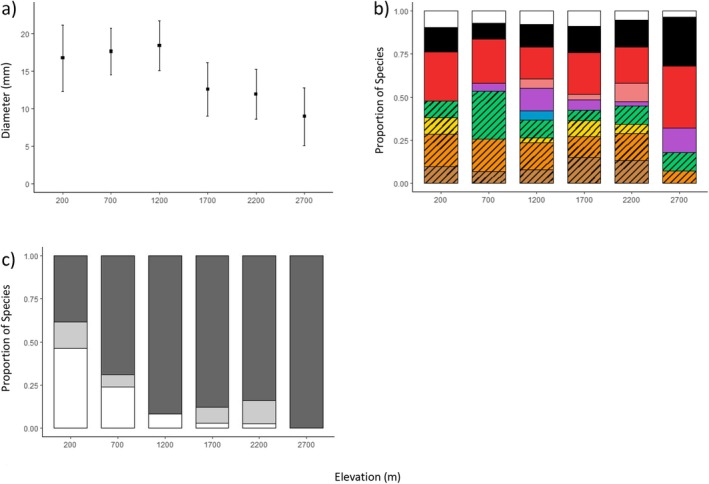
Effect of elevation on three fruit traits related to dispersal by frugivores. (a) Mean fruit diameter per fruiting plant species is represented by squares, with error bars displaying 95% confidence intervals (CIs). Letters above the points denote significant differences after adjusting for multiple comparisons using Tukey pairwise tests. (b) Proportion of fruiting plant species displaying fruits of each colour (top to bottom at 1200 m asl: White, black, red, pink, purple, blue, green, yellow, orange, brown) at each elevation. Mammal colour fruits are hatched to highlight the proportions of mammal versus bird colour fruits. (c) Proportion of fruiting plant species bearing cauliflorous fruits (white bars), ramiflorous fruits (dark grey bars) and a combination of both presentation types (light grey bars) at each elevation.

**FIGURE A1:**
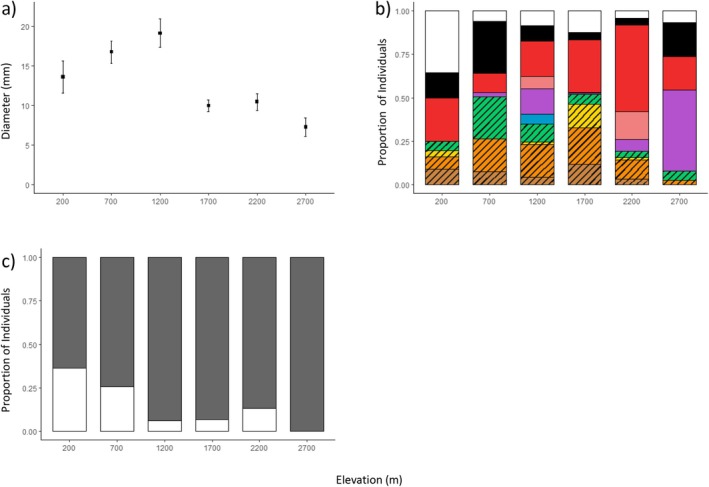
Effect of elevation on four fruit traits related to dispersal by frugivores, here weighted by plant individual. (a) Mean fruit diameter per fruiting plant is represented by squares, with error bars dispalaying 95% confidence intervals. Letters above points denote significant differences after adjusting for multiple comparisons using Tukey pairwise tests. (b) Proportion of individual fruiting plants displaying fruits of each colour (top to bottom: White, black, red, pink, purple, blue, green, yellow, orange, brown) at each elevation. ‘Mammal colour’ fruits are hatched to highlight the proportions of mammal versus bird colour fruits. (c) Proportion of fruiting plants bearing cauliflorous fruits (white bars) and ramiflorous fruits (dark grey bars) at each elevation.

**FIGURE A2:**
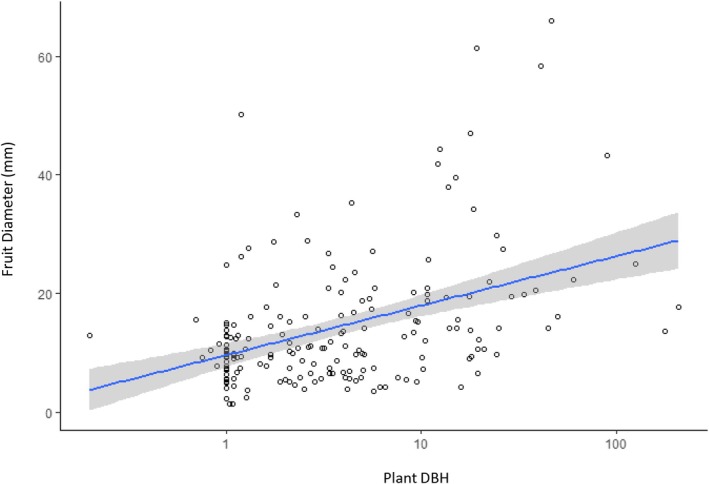
Relationship between plant diameter at breast height (DBH) and fruit diameter for all plant species recorded along the elevational gradient. Each circle represents mean DBH and fruit diameter values for a single species. The x‐axis is log‐transformed for display purposes. The blue line represents a line of best fit, with grey bars representing 95% confidence intervals. R^2^ is 0.064.

Mean fruit diameter showed a significant decrease towards higher elevations when weighted both by species (*p* < 0.01; Figure 2a) and individual fruiting plants (*p* < 0.01; Figure A1a). Fruit diameter was also positively correlated with fruiting plant DBH (*p* < 0.01; Figure A2).

### 3.2 Fruit Colour and Elevation

Fruit colour when divided into mammal versus bird colour showed a significant trend—the proportion of species bearing bird colour fruits increased with elevation (*p* < 0.01, Figure 2b and Table 1). A similar trend was observed for individual‐weighted data (*p* < 0.01, Figure A1b), although a high number of plant individuals displayed bird colour fruits at 200 m, primarily attributable to white fruits (which may also be attractive to mammals). Other fruit colours showed differing patterns depending on whether weighted by species or individual, although most colours were represented at all or nearly all elevations. Using species‐weighted data, most individual fruit colours did not show strong elevational trends (Table 1 and Table A2), although richness and abundance of species bearing green fruits peaked at 700 m and purple fruits at 2700 m.

**TABLE 1:** Results of generalised linear models testing the effect of elevation on fruit diameter, fruit colour (both for individual colours and colour category, that is, colours grouped according to bird vs. mammal dispersal syndromes) and presentation type (ramiflorous vs. cauliflorous) for fruiting plant species along the Mt. Wilhelm elevational gradient.

Parameter	Category	Deviance	*p*	Significant pairwise interactions
Fruit diameter		2338.1	< 0.01	700–2700; 1200–2700
Colour	Red	9.89	0.08	None
	Orange	11.99	0.03	None
	Yellow	21.69	< 0.01	None
	Green	21.79	< 0.01	700–1700; 700–2200
	Blue	8.62	0.13	None
	Purple	45.47	< 0.01	700–2700; 1700–2700; 2200–2700
	Pink	37.13	< 0.01	1700–2200
	Brown	18.35	< 0.01	None
	Black	15.68	< 0.01	1700–2700
	White	2.7	0.75	None
Colour category		35.73	< 0.01	700–2700; 1200–2700; 1700–2700
Presentation	Cauliflorous	36.79	< 0.01	None
	Ramiflorous	55.09	< 0.01	200–700; 200–1200; 200–1700
				200–2200; 200–2700; 700–2700

*Note:* Significant (*p* < 0.05) individual pairwise interactions between elevations are presented, after correcting for multiple comparisons using Tukey pairwise comparisons. Significance values for all pairwise comparisons are displayed in full in Table A2.

### 3.3 Fruit Presentation and Elevation

Fruit presentation showed a clear trend with elevation: less than 40% of fruiting species were exclusively ramiflorous at 200 m, increasing to 100% at 2700 m (*p* < 0.01; Figure 2c). We observed a similar increase with elevation in individual‐weighted data (*p* < 0.01; Figure A1c).

### 3.4 Fruit Syndromes

**FIGURE 3:**
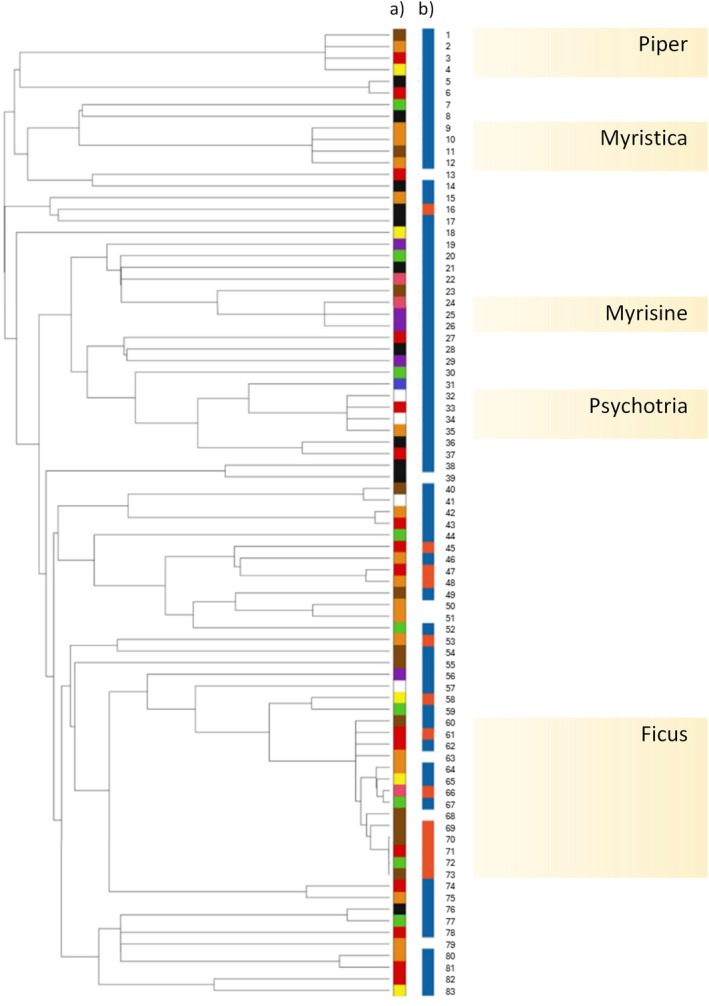
Phylogenies of the 83 fruiting plant species for which phylogenetic data was available, including fruit colour (a) and the presentation method of fruits (b). In (a), colours of squares represent the predominant colours of the fruits of that species (see Figure 2b for colour list). In (b), blue squares represent ramiflorous fruiting species, while orange squares represent cauliflorous species. Species without squares attributed to them indicate a combination of ramiflory and cauliflory within a single species. Numbers correspond to species names listed in Table A3. Prominent genera (represented by more than two species) are displayed on the right.

Phylogenetic analysis on the categorical traits of fruit colour and presentation showed broadly contrasting patterns. In general, fruit colour did not differ from random expectations, suggesting that fruit colour is not phylogenetically constrained and rather subject to selection (Figure 3a and Table A4). Of all colour categories, only brown fruits appear to be phylogenetically clustered (SES.MPD = −2.21, *p* = 0.03). Fruit presentation, however, showed evidence of significant phylogenetic clustering: cauliflorous fruits were clustered significantly more than expected under null models (SES.MPD = −7.12, *p* < 0.01; Figure 3b). For the continuous trait of fruit diameter, a low phylogenetic signal indicated a lack of phylogenetic clustering (λ = 0.29, *p* = 0.02).

**FIGURE 4:**
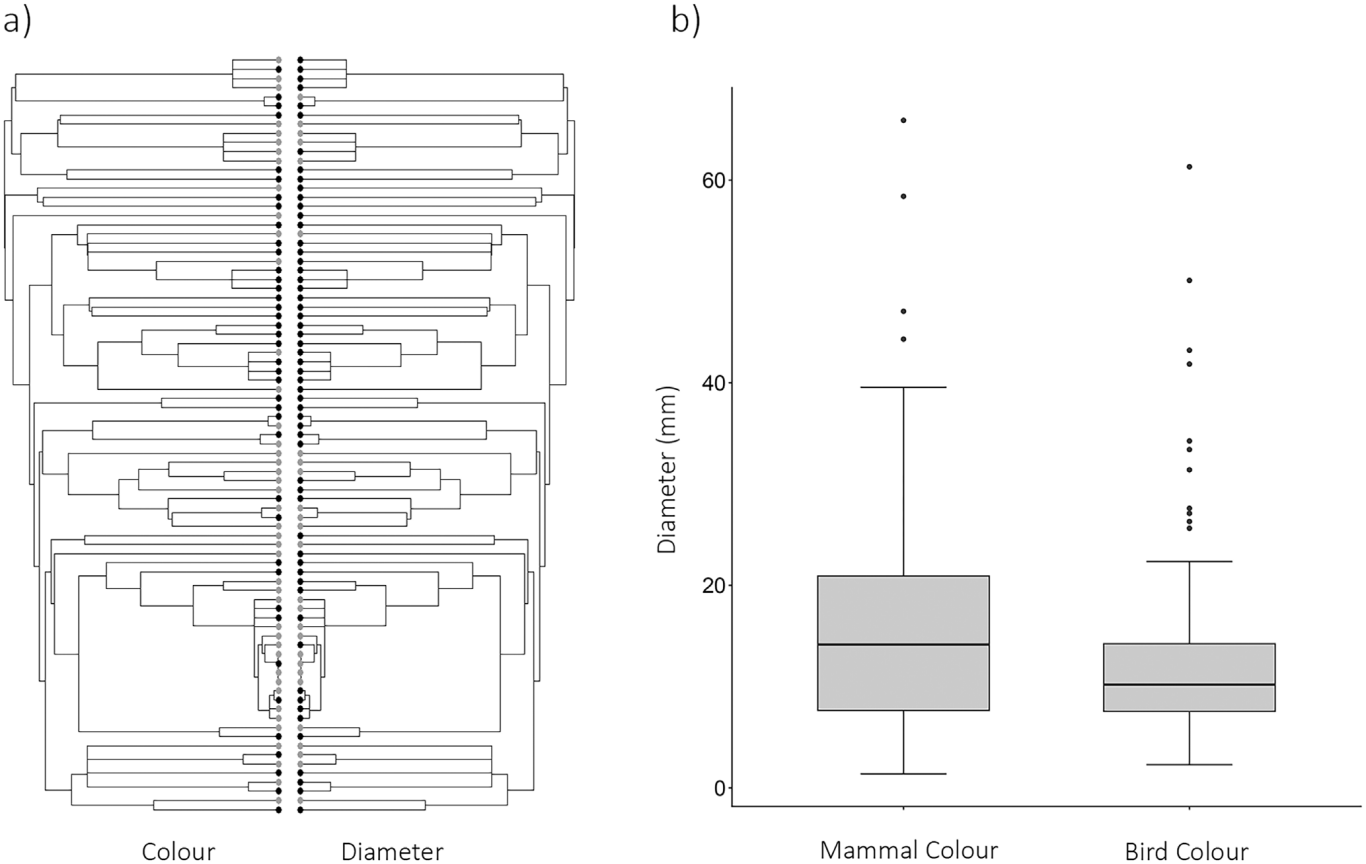
Relationships between fruit colour and diameter for all fruiting plant species and morpho‐species recorded along the Mt. Wilhelm gradient. (a) Visualisation of data from Pagel's likelihood ratio test between fruit colour (left phylogeny) and diameter (right phylogeny), where each trait is represented by two binary categories. For colour, grey circles represent ‘mammal colour’ (green, yellow, orange and brown) fruits and black circles ‘bird colour’ (red, pink, purple, blue, black and white) fruits. For mean diameter, grey circles represent ‘large‐fruited’ species and black circles ‘small‐fruited’ species. (b) Relationship between fruit colour category (as above) and fruit diameter, this time displayed as a continuous variable. Grey boxes span the first and third quartiles of fruit diameter and the horizontal line within each box represents the median diameter. Vertical lines indicate maximum and minimum observations falling within 1.5 times the interquartile range. Remaining observations are displayed as black circles.

Both phylogenetic and non‐phylogenetic analyses revealed significant relationships between fruit colour and size. We observed a significant phylogenetic association between ‘large’ and ‘small’ fruits, and mammal colour and bird colour fruits, respectively (likelihood ratio = 14.33, *p* < 0.01; Figure 4a), showing evidence of correlated evolution of fruit colour and size. Regardless of phylogeny, we found mammal colour fruits to have significantly greater median diameter than bird colour fruits (median_mammal colour_ = 14.06 mm, median_bird colour_ = 10.2 mm, W = 6026, *p* < 0.01; Figure 4b) and to show a greater variation in size (Fligner‐Killeen median χ^2^ = 18.04, *p* < 0.01; Figure 4b). The mean diameters of each fruit category were 17 mm (mammal colour) and 12.8 mm (bird colour).

## 4 Discussion

In this pilot study, we examined three key fruit traits—size (represented by diameter), colour and presentation—associated with dispersal by vertebrate frugivores along the elevational gradient of Mt. Wilhelm in Papua New Guinea. To our knowledge, this work provides the first analysis of fruit traits across a tropical fruiting plant community spanning an elevational gradient and should serve as a starting point for further research into fruit traits across elevation at regional or global scales. By analysing these traits, we also tested the dispersal syndromes hypothesis, which posits that specific fruit characteristics are shaped by the preferences of their primary dispersers.

### 4.1 Fruit Size and Elevation

We found mean fruit diameter to decrease towards higher elevations, after a plateau between 200 and 1200 m, partially supporting our first hypothesis. Our result mirrors a trend observed by Almeida‐Neto et al. (2008), who found fruit size to be lower at higher elevations across 135 sites in the Brazilian Atlantic rainforest. A few potential factors could explain the observed pattern: (i) A decrease in productivity with decreasing temperature at higher elevations could limit the production of large fruits. (ii) Fruit size could be constrained by plant size or other developmental constraints such as leaf size, which in turn might decrease with elevation. (iii) A lack of large‐gaped frugivores at high elevations, perhaps driven by environmental constraints on frugivore size, could prevent the seed dispersal of large‐fruited plants. These hypotheses are not mutually exclusive, and the reality is likely a result of multiple interacting factors. Here we briefly discuss each one.

As temperature decreases consistently with elevation—recorded by dataloggers at the study site (Sam et al. 2019)—one might expect fruit size to show a corresponding steady decline if temperature was the dominant factor influencing fruit size. However, Almeida‐Neto et al. (2008) found that differences in fruit size with elevation were primarily not attributable to temperature. Specifically, low‐elevation communities had greater mean fruit diameter than higher‐elevation communities experiencing similar annual temperatures (i.e., those from warmer areas). The differing patterns of fruit size decrease versus temperature decrease with elevation at Mt. Wilhelm further suggest that temperature cannot fully explain the pattern in fruit size observed here. Broader productivity factors, including rainfall, may help to account for fruit size trends (Trethowan et al. 2023), but while Sam et al. (2024) report higher annual precipitation at 2700 m than at 200 m, we lack precipitation data for intermediate sites to adequately test the relationship. Other abiotic factors such as light intensity and soil type could also potentially influence fruit size—for example, Parolin (2000) found seed size to be influenced by varying soil nutrients in Amazonian floodplains. Overall though, the potential influences of abiotic factors on fruit size in the context of elevation is an understudied area (Trethowan et al. 2023).

Regarding the potential influence of plant size on fruit size, we found a positive correlation between fruit diameter and fruiting plant DBH; yet elevation still explained variation in fruit size even after accounting for DBH. Qi et al. (2014) found elevational differences in seed size to be strongly related to plant height in Tibet, but when focusing on zoochorous (animal‐dispersed) seeds, no significant elevation pattern in seed mass was found. Other potential developmental constraints on fruit size have been hypothesised. Herrera (2002) predicted that fruit size could be related to leaf size because both may share developmental pathways. However, sizes of zoochorous fruits in a Colombian forest were unrelated to leaf size, in contrast to fruits with different dispersal modes (Stevenson et al. 2023). Such patterns tentatively support the idea that selection by frugivores could disrupt the developmental relationships between fruit size and other plant traits.

The relationship between fruit size and disperser size at the community level is complex, although an association between the two groups has been noted by several studies (Burns 2013; Dehling et al. 2014; Muñoz et al. 2017; Bender et al. 2018). Our data, which show a low‐elevation plateau in fruit size followed by a decline towards mid‐elevations, is consistent with a change in the avian frugivore community observed between lowland and highland forest. Sam et al. (2019) documented a mid‐elevational shift in the distribution of the large‐gaped frugivores, such as the Papuan hornbill *Rhyticeros plicatus* and the majority of pigeon species, which are primarily found between 200 and 1200 m asl. Furthermore, species richness of bats (which are not gape‐limited and may consume larger fruits than birds) is known to decline with elevation in Papua New Guinea (McCain 2007) and in the study area specifically (Sivault et al. 2023). Hazell et al. (2023) noted a decline in bat frugivory with elevation here, as has also been noted elsewhere (McCain 2007; Almeida‐Neto et al. 2008). While noteworthy, these trends do not provide a causal link between fruit size and disperser size across elevations. Large‐scale datasets on fruit and disperser traits and their interactions from across multiple elevational gradients are encouraged, comparable to global studies such as those conducted by Sinnott‐Armstrong et al. (2018) and McFadden et al. (2022), which have found evidence of fruit–frugivore size matching across climatic gradients.

### 4.2 Fruit Colour and Elevation

Prevalence of combined bird colour fruits (red, pink, purple, blue, black, white) increased with increased elevation, specifically driven by their dominance at 2700 m. This trend tentatively supports our second hypothesis, which states that the increased relative importance of avian frugivory at higher elevations would be associated with higher prevalence of these fruit colours, in line with the dispersal syndromes hypothesis (see below). As with fruit size, the relative prevalence of bird versus mammal colour fruits across elevations requires further research in order to reveal any causal relationships, as such studies are currently lacking. Meanwhile we found few clear trends in specific fruit colours along the elevational gradient, although green, purple and black fruits each showed some significant variations in prevalence. A recent study found no relationship between fruit colour and specific avian colour preferences along the same gradient (Hazell et al. 2023), suggesting birds are exerting little selective pressure on specific fruit colours.

Fruit colour may also reflect an adaptation to abiotic factors (Burns 2015; Valenta et al. 2018). Anthocyanins, which are responsible for blue, deep red, purple and black colours in fruit, may serve an important role in protecting plants from abiotic stressors such as temperature, drought, salinity and UV‐B radiation (Kaur et al. 2023; Dellinger et al. 2025). Thus, their presence in other plant tissues and in fruits may be coupled, meaning fruit colours characterised by high anthocyanin content could simply be a by‐product of this effect (Stournaras and Schaefer 2017). In a global latitudinal study, Dellinger et al. (2025) found red fruits to be more dominant in higher (colder) latitudes, aligning with the idea that pigmentation could be aiding in thermoregulation—although this was not supported by black fruits which were common in warm environments. The scarcity of individual colour trends in our study precludes any strong conclusions on elevational adaptations of fruit colour to abiotic factors. However, we note that the combined dominance of red, purple and black fruits at 2700 m (where the mean annual temperature is below 15°C) offers a potential explanation in terms of adaptation to cold stress via anthocyanin pigmentation. Anthocyanins are also known to increase after exposure to UV‐B radiation, possibly to prevent photoinhibition (Sinnott‐Armstrong et al. 2021). This supports the idea that higher elevation forests with more open canopies could promote an increased prevalence of red, purple and black fruits.

### 4.3 Fruit Presentation and Elevation

Elevation had a significant effect on the presentation method of fruits. Cauliflorous fruits were relatively common at 200 m but steadily declined with elevation and were absent at 2700 m, supporting our third hypothesis. This pattern aligns with the sharp decline in bat frugivory with increasing elevation. While birds typically require a branch on which to perch while feeding, several bat species (including the flying foxes *Pteropus*) are known to preferentially forage on fruits accessible in open spaces, including cauliflorous fruits (Whittaker and Jones 1994; Korine et al. 2000). The above‐mentioned elevational decrease in bat species richness (Sivault et al. 2023) and bat frugivory (Hazell et al. 2023) in the study area could potentially, therefore, be at least partially related to the parallel decline in cauliflorous fruits, leading to a relatively greater importance of avian frugivores at higher elevations. However, arboreal mammals, including rodents and marsupials, do not show a similar decline across the elevations in this study (Hazell et al. 2023; Vejmělka 2025). Arboreal mammals may easily access fruit presented on the main stem of a fruiting plant and thus could potentially support dispersal of cauliflorous fruits at higher elevations. Further study on mammalian frugivory of cauliflorous fruits is thus necessary to determine any potential adaptive effects of frugivory on cauliflory across elevations.

It should be noted that cauliflory showed significant phylogenetic clustering in this study, meaning the effects of phylogenetic inertia cannot be discounted in structuring the observed patterns. Additionally, as with fruit size and colour, cauliflory may be influenced by adaptive factors other than dispersers. For example, Harrison et al. (2012) suggest that in figs, a prominent cauliflorous genus in our study, fruits being borne close to the trunk could confer evolutionary benefits via recovery of nutrients from dropped male figs after the pollinating wasps have emerged.

### 4.4 Fruit Syndromes

Our results support the dispersal syndrome hypothesis. Across elevations, we demonstrated a clear relationship between fruit colour category (the groups of colours commonly attributed to dispersal by either mammals or birds) and fruit size, supporting our fourth hypothesis. We found mammal colour fruits (brown, green, yellow and orange) to be larger than bird colour fruits (red, pink, purple, blue, black and white) and to show a greater variation in size. Both of these factors are consistent with adaptation to dispersal by mammalian versus avian frugivores. Our results mirror global patterns observed by Sinnott‐Armstrong et al. (2018), who found strong evidence across a global fruit trait database that larger fruits tend to be brown, green, yellow and orange in colour. In a latitudinal parallel of our observed elevational patterns, they found such larger, duller‐coloured fruits to be more prevalent at tropical latitudes where mammalian frugivory is relatively more important than in temperate zones, where smaller and more brightly coloured (especially red) fruits dominated.

The association between fruit colour category and size appears across separate clades within the phylogeny of fruiting plants, indicating correlated evolution of size and colour category and supporting our fifth hypothesis. This occurs despite there being no significant evidence of phylogenetic clustering of individual fruit colours (except brown), which are known to be evolutionarily labile. Consistent with our findings, studies from the Brazilian Atlantic Forest (Cazetta et al. 2012) and from a subtropical Andean forest (Ordano et al. 2017) found little to no phylogenetic signal in fruit colour, despite its presence in other fruit traits. On a broader scale, Stournaras et al. (2013) found minimal evidence of phylogenetic constraints on fruit colour at both local and global scales, despite fruit colour being limited by other factors such as chemical constraints. The fact that individual fruit colours are spread across the phylogeny, but that colour still correlates with fruit size at the broader level of colour category (mammal vs. bird), supports the hypothesis that fruit colours have adapted independently to dispersal by different frugivore guilds. Thus, we discount the hypothesis of phylogenetic inertia as the means of explaining observed fruit colour/size combinations.

## 5 Summary

We observed that fruit traits related to mammalian frugivory (large size, dull colours, ramiflorous presentation) decreased at higher elevations, while traits related to avian frugivory generally increased. Due to the relatively small scale of this pilot study, we are cautious not to ascribe elevational trends in fruit traits directly to adaptation to dispersers. We encourage research across a range of elevational gradients, and in particular, making use of long‐term fruit trait datasets, to further explore the nature of these relationships. The roles of certain abiotic variables could also be important and should be factored into any further investigations. Nevertheless, a significant relationship between fruit size and colour categories across sites, independent of phylogeny, supports the dispersal syndromes hypothesis. The fruit traits investigated are clearly important in determining frugivory by different guilds, influencing seed dispersal and ultimately plant community assembly. The shifting distributions of plants and frugivores resulting from climate change have the potential to disrupt existing relationships and should be another focus of further study.

## Author Contributions

**Richard J. Hazell:** conceptualization (equal), data curation (equal), formal analysis (equal), investigation (equal), methodology (equal), project administration (equal), resources (equal), visualization (equal), writing – original draft (equal), writing – review and editing (equal). **Graham S. Kaina:** investigation (equal), methodology (equal), project administration (equal), resources (equal), writing – review and editing (equal). **Katerina Sam:** conceptualization (equal), funding acquisition (equal), methodology (equal), project administration (equal), resources (equal), supervision (equal), visualization (equal), writing – review and editing (equal). **Daniel Souto‐Vilarós:** conceptualization (equal), data curation (equal), formal analysis (equal), methodology (equal), resources (equal), writing – review and editing (equal). **Wulan Koagouw:** formal analysis (equal), project administration (equal), visualization (equal), writing – review and editing (equal). **Vojtech Novotny:** conceptualization (equal), funding acquisition (equal), methodology (equal), writing – review and editing (equal). **Mika R. Peck:** funding acquisition (equal), methodology (equal), project administration (equal), supervision (equal), writing – review and editing (equal). **Alan J. A. Stewart:** funding acquisition (equal), methodology (equal), project administration (equal), supervision (equal), writing – review and editing (equal).

## Funding

This work was supported by the UK Darwin Initiative (19‐008). R.H. would like to thank the Sir Richard Stapley Educational Trust for its generous financial support. V.N. was funded by Praemium Academiae from CAS. K.S. acknowledges Czech Science Foundation Junior Star 22‐17593M.

## Conflicts of Interest

The authors declare no conflicts of interest.

## Data Availability

All data and scripts supporting the findings of this study are openly available in the FigShare digital repository at the following DOI: https://doi.org/10.6084/m9.figshare.30043075.v1. This collection includes: Sample locations and abundance and trait data of fruiting plants and fruits; hierarchical analysis of plant phylogenies, likelihood ratio tests for fruit syndromes, R code for calculation of statistical analyses and creation of plots, plus associated files.

## Appendix A

Note on plant phylogeny: The phylogeny used in this study is adapted from Open Tree of Life version 9.1, following hierarchical analysis with individual phylogenies constructed for major clades and can be found at https://github.com/FePhyFoFum/big_seed_plant_trees/releases (labelled ‘ALLOTB’), in addition to the Figshare repository for project data.

**TABLE A1:** List of fruiting plant species and morpho‐species recorded at each elevation, together with information on fruit traits.

Elevation	species	Fruit diameter	Seed proportion	Presentation	Colour
200 m	Aglaia sp. 1	21.4	0.452556818	NA	Orange
200 m	*Allophylus cobbe*	6.466666667	0.397959184	C	Red
200 m	ANNONACEAE sp. 1	13.98	0.537081024	C	Black
200 m	Artocarpus lakoocha	7.228571429	NA	C	Yellow
200 m	Casearia clutiifolia	16.05	0.405228758	NA	Orange
200 m	Erythrospermum candidum	9	0.269230769	NA	Green
200 m	Ficus congesta	38	0.325098039	C	Brown
200 m	Ficus sp. 5	20.16	0.208609272	C	Green
200 m	Ganalium sp. 1	22.06666667	0.430965682	R	Black
200 m	Harpullia ramiflora	22.33333333	0.07753051	C	Red
200 m	Leea indica	11.17142857	0.191366906	C + *R*	Black
200 m	Litsea collina	14.25714286	0.493804956	NA	Red
200 m	Micromelum minutum	5.9	0.361111111	NA	Orange
200 m	Myristica sp. 3	20.5	0.139720559	NA	Yellow
200 m	Myristica sp. 4	19.5	0.452399685	NA	Brown
200 m	Pittosporum sinuatum	11.5	NA	R	Red
200 m	Psychotria leptothyrsa	11.94	0.25	R	Red
200 m	Psychotria sp. 1	5.533333333	0.459016393	R	White
200 m	Psychotria sp. 2	12.91666667	0.176056338	R	White
200 m	SAPINDACEAE sp. 1	25.63	0.5944	C + *R*	Red
200 m	Wenzelia dolichophylla	35.225	0.139579148	NA	Orange
700 m	Aglaia sp. 2	13.82857143	0.59268762	R	Red
700 m	ANNONACEAE sp. 2	9.43	0.441558442	R	Red
700 m	Archidendron aruense	19.3	0.114798206	C	Orange
700 m	Archidendron sp. 1	27.1	0.070609812	C	Red
700 m	Ardisia imperialis	6.5	0.454545455	R	Purple
700 m	Ardisia sp. 2	11.06086957	0.493767313	R	Green
700 m	Ardisia sp. 1	10.87777778	0.300306435	R	Green
700 m	Clerodendrum sp. 1	9.8	0.574468085	R	Black
700 m	Cryptocarya sp. 1	11.26666667	0.513368984	R	Green
700 m	Cupaniopsis acuticarpa	20.975	0.375780275	R	Orange
700 m	Cupaniopsis sp. 1	13.7	0.15503876	R	Green
700 m	Ficus badiopurpurea	5.1	0.235294118	R	Green
700 m	Ficus bernaysii	18.8015873	0.310492776	C	Green
700 m	Ficus hahliana	15.4	0.217252396	C	Green
700 m	Ficus morobensis	39.55	0.129220509	C	Brown
700 m	Ficus pungens	6.555	0.367454068	C	Brown
700 m	Ficus saccata	18.3	0.125364431	NA	Orange
700 m	Ficus sangumae	13.8	0.218023256	C	Red
700 m	Ficus sp. 3	29.83333333	NA	R	Brown
700 m	Ficus sp. 4	47.05	0.258940112	C	Green
700 m	Ficus wassa	14.7	0.317567568	C	Red
700 m	Glochidion angulatum	5.8	0.210526316	R	Green
700 m	Gymnacranthera paniculata	20.85	0.095317726	R	Orange
700 m	Harpullia longipetala	23.63131222	0.215984645	C + *R*	orange
700 m	Harpullia sp. 1	24.5	0.068085106	R	Green
700 m	Harpullia sp. 2	20.16	0.030376671	C	Green
700 m	Homalanthus novoguineensis	5.9	0.125	R	Red
700 m	Kibara sp. 2	9.7	0.481927711	R	Black
700 m	LAMIACEAE sp. 1	8.2	0.285714286	R	Red
700 m	Leucosyke australis	14.7	0.255093003	R	White
700 m	Leucosyke sp. 1	10.57	NA	R	White
700 m	Magnolia tsiampacca	13.7	0.691442629	R	Orange
700 m	Medinilla crassinervia	17.75714286	0.41503268	C + *R*	Purple
700 m	Melastoma sp. 1	9.3	NA	R	Red
700 m	Myristica subalulata	5.466666667	0.466666667	R	Orange
700 m	Pandanus kaernbachii	7.85	0.152091255	R	Orange
700 m	Pittosporum sinuatum	9.2	NA	R	Red
700 m	Planchonella sp. 1	61.32	0.562448582	R	Black
700 m	Popowia pisocarpa	13.51333333	0.235361653	C + *R*	Black
700 m	Psychotria micrococca	6.78	0.339233038	R	White
700 m	Sterculia schumanniana	26.73333333	0.174298045	R	Green
700 m	Syzygium gonathantum	50.1	0.246326476	R	Red
700 m	Syzygium sp. 1	27.6	0.238843931	R	Red
1200 m	Aglaia tomentosa	12.36666667	0.275862069	R	Green
1200 m	Annesijoa novoguineensis	19.58	0.085042521	R	Yellow
1200 m	ANNONACEAE sp. 3	11.76666667	0.153488372	R	Purple
1200 m	Ardisia imperialis	6.891666667	0.680208333	R	Black
1200 m	Ardisia lanceolata	14.5	0.293144208	R	Pink
1200 m	Ardisia sp. 2	10.75555556	0.430156472	R	Red
1200 m	Ardisia sp. 1	9.85	0.684039088	R	Red
1200 m	Areca sp. 1	10.5125	0.732732733	R	Red
1200 m	Caryota rumphiana	14.23	NA	C	Black
1200 m	Cyclophyllum brevipes	7.3	0.416666667	R	Blue
1200 m	*Cyrtandra erectiloba*	12.45	0.56119403	R	White
1200 m	FAGACEAE sp. 1	17.8125	NA	R	Black
1200 m	Garcinia maluensis	28.8	0.532967033	R	Orange
1200 m	Harpullia longipetala	18.72857143	0.563312229	NA	Red
1200 m	Harpullia sp. 3	13.22	0.581772334	C	Red
1200 m	Kibara sp. 1	9.06	0.47826087	R	Black
1200 m	Magnolia tsiampacca	24.93333333	NA	R	Green
1200 m	Medinilla crassinervia	15.1	NA	C	Green
1200 m	Melastoma sp. 1	16.15	0.320366133	R	Pink
1200 m	Myristica fatua	58.4	0.027943934	R	Orange
1200 m	Myristica filipes	65.9	0.224500898	R	Brown
1200 m	Myristica sp. 1	16.375	0.519174041	R	Orange
1200 m	Myristica sp. 2	19.8	0.319218241	R	Brown
1200 m	Myristica subalulata	27.5	NA	R	Brown
1200 m	Planchonella macropoda	43.2	0.31828784	R	Black
1200 m	Prunus dolichobotrys	24.9	NA	R	Orange
1200 m	Rapanea involucrata	10.3	0.46875	R	Red
1200 m	RUBIACEAE sp. 1	9.45	0.254901961	R	Purple
1200 m	RUBIACEAE sp. 2	11.42	0.460893855	R	White
1200 m	RUBIACEAE sp. 3	9.842857143	0.088328076	R	Purple
1200 m	RUBIACEAE sp. 3	10.4826087	0.270524582	R	Blue
1200 m	RUBIACEAE sp. 3	10.57142857	0.270306258	R	Purple
1200 m	SOLANACEAE sp. 1	29	0.524161074	NA	Orange
1200 m	Solanum sp. 1	12.93333333	NA	R	Orange
1200 m	Solanum sp. 2	15.3	0.646586345	R	Red
1200 m	Terminalia sp. 1	31.1	0.687538493	R	Green
1200 m	ULMACEAE sp. 1	5.031578947	NA	R	White
1200 m	Ziziphus angustifolia	12.7125	0.568421053	R	Purple
1700 m	Ardisia sp. 4	26.3	NA	R	Pink
1700 m	Canarium sp. 1	9.8	0.224299065	R	Black
1700 m	Chisocheton sp. 1	33.4	0.197997775	R	Red
1700 m	Ficus arfakensis	16.85	0.41285297	R	Brown
1700 m	Ficus hahliana	20.83333333	0.343481138	C + *R*	Red
1700 m	Ficus sangumae	12.42857143	0.427622842	C	Yellow
1700 m	Ficus subulata	4.4	0.147058824	R	Yellow
1700 m	Ficus trachypison	14.15	0.324324324	R	Orange
1700 m	Ficus trichocerasa	12.25	0.201834862	C + *R*	Red
1700 m	Ficus wassa	9.8	0.24	C + *R*	Red
1700 m	Kibara coriacea	10.23333333	0.421276596	R	Purple
1700 m	Lepidopetalum comesperma	9.8	0.478021978	R	Green
1700 m	Melicope elleryana	17.4	0.026359143	R	Brown
1700 m	Myristica filipes	44.3	NA	R	Brown
1700 m	Osmoxylon sp. 1	1.433333333	NA	R	Orange
1700 m	Osmoxylon sp. 2	5.7	0.625	R	Black
1700 m	Piper interruptum	7.468292683	NA	R	Red
1700 m	Piper macropiper	2.4	NA	R	Brown
1700 m	Piper recessum	6.775	NA	R	Yellow
1700 m	Piper sp. 1	8.5	NA	R	Red
1700 m	Piper sp. 2	9.2	NA	R	Red
1700 m	Piper sp. 3	10.4	NA	R	Orange
1700 m	Pittosporum sp. 1	10.53636364	0.620147874	R	Orange
1700 m	Psychotria micrococca	9.85	0.548148148	R	White
1700 m	Psychotria sp. 3	4.6	0.5	R	White
1700 m	RHAMNACEAE sp. 1	10.2	0.507246377	R	Black
1700 m	RUBIACEAE sp. 4	4.014285714	0.511415525	R	White
1700 m	Saurauia conferta	29.66666667	NA	R	Green
1700 m	Smilax nova‐guineensis	13	0.325581395	R	Black
1700 m	Sterculia schumanniana	15.54285714	0.54754289	R	Black
1700 m	*Tabernaemontana pandacaqui*	10.2	NA	R	Red
1700 m	VITACEAE sp. 1	2.283333333	NA	R	Purple
1700 m	Xanthophyllum papuanum	10.6	0.375838926	R	Brown
2200 m	ANNONACEAE sp. 1	28.9	0.126213592	R	Green
2200 m	Ardisia sp. 2	9	0.346153846	R	Pink
2200 m	Ardisia sp. 1	7.42	0.233918129	R	Black
2200 m	Astronidium sp. 1	12.8	0.172506739	R	White
2200 m	*Cayratia trifolia*	9.5	0.26	R	Black
2200 m	*Cyrtandra erectiloba*	10.7	NA	R	Green
2200 m	Decaspermum alpinum	5.05	0.588235294	R	Orange
2200 m	Decaspermum forbesii	5.2	0.25	R	Orange
2200 m	Embelia cotinoides	12.5	NA	R	Pink
2200 m	Ficus iodotricha	41.84	NA	C	Red
2200 m	Ficus microdictya	19.8	NA	NA	Brown
2200 m	Ficus sangumae	15.55	0.291666667	C + *R*	Pink
2200 m	Ficus sp. 1	16.05	0.138790036	R	Green
2200 m	Ficus sp. 2	22.35	0.259932245	R	Red
2200 m	Ficus wassa	7.483125	0.399505728	C + *R*	Pink
2200 m	Hydriastele sp. 1	14.22	0.493333333	C + *R*	Red
2200 m	Ichnocarpus frutescens	9.4	0.266666667	R	Black
2200 m	Kibara coriacea	10.73333333	0.652892562	R	Black
2200 m	Maesa haplobotrys	3.966666667	0.333333333	R	Brown
2200 m	Medinilla crassinervia	5.2	NA	C + *R*	White
2200 m	Medinilla lorentziana	5.5	0.25	R	Brown
2200 m	Melastoma sp. 2	7.5	0.428571429	R	Green
2200 m	Melicope sp. 1	16.575	0.018034265	R	Yellow
2200 m	Myrsine womersleyi	5.351443001	0.366424337	R	Purple
2200 m	Osmoxylon sp. 1	1.385714286	NA	R	Orange
2200 m	Pandanus sp. 1	9.42	0.042666667	C + *R*	Red
2200 m	Piper celtidiforme	12.8	NA	R	Orange
2200 m	Piper macropiper	3.7544	NA	R	Red
2200 m	Piper sp. 5	7.1	NA	R	Orange
2200 m	Pittosporum sp. 1	7.74375	NA	R	Red
2200 m	Polyosma cunninghamii	8.1	0.125	R	Black
2200 m	Psychotria murmurensis	5.2	NA	R	Orange
2200 m	Scaevola oppositifolia	5.65	0.5	R	Black
2200 m	Smilax nova‐guineensis	10.2	0.4375	R	Brown
2200 m	Timonius sp. 1	31.4	NA	R	Red
2200 m	Tinospora cordifolia	5.6	0.363636364	R	Yellow
2200 m	Tripetalum cymosum	34.25	0.22165069	R	Red
2200 m	Zehneria mucronata	6.4	0.153846154	R	Brown
2700 m	Ardisia sp. 1	5.777192982	0.224238579	R	Black
2700 m	Ardisia sp. 3	5.625	0.347826087	R	Black
2700 m	Bhesa archboldiana	3.6	0.6	R	Red
2700 m	Breynia cernua	6.6	0.434782609	R	Black
2700 m	Decaspermum alpinum	9.3	NA	R	Red
2700 m	Elaeocarpus nymanii	14.16	0.806060606	R	Green
2700 m	Eurya tigang	9.2	NA	R	Purple
2700 m	Glochidion sp. 2	4.3	0.375	R	Red
2700 m	Glochidion sp. 1	4.22	0.396825397	R	Red
2700 m	Kibara coriacea	11.12	0.516332982	R	Black
2700 m	Kibara sp. 3	19.08	0.266677409	R	Red
2700 m	Maesa edulis	4.166666667	0.4	R	Red
2700 m	Myrsine involucrata	3.964788732	0.405444887	R	Purple
2700 m	Myrsine womersleyi	7.007894737	0.428751576	R	Purple
2700 m	Palmeria sp. 1	7.866666667	0.448275862	R	Red
2700 m	Piper sp. 4	16.2	NA	R	Orange
2700 m	Polyosma cunninghamii	8.633333333	0.419548872	R	Purple
2700 m	Prunus oligantha	13.15714286	0.481472081	R	Red
2700 m	Prunus sp. 1	14.7	0.452631579	R	Black
2700 m	Psychotria multicostata	9.990909091	0.299354244	R	White
2700 m	RUBIACEAE sp. 1	7.8	NA	R	Orange
2700 m	Saurauia sp. 1	12.01428571	0.291233284	R	Green
2700 m	Solanum sp. 1	8.6	0.625	R	Red
2700 m	Steganthera sp. 1	9.24	0.631141345	R	Black
2700 m	Streblus glaber	5.6	0	R	Green
2700 m	Streblus sp. 1	7.425	0.379310345	R	Black
2700 m	Zygogynum haplopus	5.216666667	0.295081967	R	Black
2700 m	Zygogynum oligostigma	15.25	NA	R	Red

*Note:* Species means are displayed for fruit diameter and for the proportion of fruit weight attributable to seeds. NA values are given where seeds could not easily be separately weighed (e.g., compound fruits). Presentation types include cauliflorous (C), ramiflorous (R) and ‘C + *R*’ (species with individuals displaying either presentation type). NA values are given for species for which individuals displayed both types of presentation simultaneously. The mode fruit colour is also listed for each species.

**TABLE A2:** Results of Tukey pairwise comparisons from generalised linear models testing the effect of elevation on different fruit traits (left column).

Parameter	Elevation pairs	Estimate	SE	z‐ratio	*p*
Fruit diameter	200–700	−0.885	2.76	−0.321	0.9995
	200–1200	−1.672	2.81	−0.594	0.9915
	200–1700	4.148	2.89	1.436	0.7051
	200–2200	4.819	2.81	1.713	0.5234
	200–2700	7.781	2.99	2.605	0.0961
	700–1200	−0.787	2.3	−0.342	0.9994
	700–1700	5.032	2.39	2.101	0.2865
	700–2200	5.703	2.3	2.475	0.1314
	**700–2700**	**8.665**	**2.51**	**3.448**	**0.0075**
	1200–1700	5.819	2.46	2.363	0.1693
	1200–2200	6.49	2.37	2.734	0.0687
	**1200–2700**	**9.452**	**2.58**	**3.667**	**0.0033**
	1700–2200	0.671	2.46	0.272	0.9998
	1700–2700	3.633	2.66	1.366	0.7473
	2200–2700	2.962	2.58	1.149	0.8606
Seed proportion	200–700	0.17847	0.599	0.298	0.9997
	200–1200	−0.34155	0.611	−0.559	0.9936
	200–1700	−0.20215	0.662	−0.305	0.9996
	200–2200	0.17547	0.648	0.271	0.9998
	200–2700	−0.33951	0.645	−0.527	0.9951
	700–1200	−0.52003	0.51	−1.019	0.9117
	700–1700	−0.38062	0.57	−0.668	0.9854
	700–2200	−0.003	0.554	−0.005	1
	700–2700	−0.51799	0.55	−0.942	0.9355
	1200–1700	0.13941	0.582	0.239	0.9999
	1200–2200	0.51703	0.567	0.912	0.9437
	1200–2700	0.00204	0.563	0.004	1
	1700–2200	0.37762	0.621	0.608	0.9905
	1700–2700	−0.13737	0.617	−0.223	0.9999
	2200–2700	−0.51499	0.603	−0.854	0.9571
Colour category: Mammal	200–700	−0.883	0.452	−1.954	0.3692
	200–1200	−0.2311	0.486	−0.475	0.997
	200–1700	−0.6705	0.428	−1.566	0.6213
	200–2200	0.0873	0.455	0.192	1
	200–2700	1.2217	0.539	2.268	0.2072
	700–1200	0.6519	0.381	1.709	0.5257
	700–1700	0.2125	0.304	0.699	0.9821
	700–2200	0.9704	0.341	2.843	0.0509
	**700–2700**	**2.1047**	**0.446**	**4.716**	**< 0.0001**
	1200–1700	−0.4394	0.353	−1.244	0.8149
	1200–2200	0.3185	0.386	0.826	0.9629
	**1200–2700**	**1.4528**	**0.481**	**3.02**	**0.0304**
	1700–2200	0.7578	0.309	2.451	0.1391
	**1700–2700**	**1.8922**	**0.422**	**4.481**	**0.0001**
	2200–2700	1.1343	0.45	2.521	0.1178
Colour category: Bird	200–700	0.883	0.452	1.954	0.3692
	200–1200	0.2311	0.486	0.475	0.997
	200–1700	0.6705	0.428	1.566	0.6213
	200–2200	−0.0873	0.455	−0.192	1
	200–2700	−1.2217	0.539	−2.268	0.2072
	700–1200	−0.6519	0.381	−1.709	0.5257
	700–1700	−0.2125	0.304	−0.699	0.9821
	700–2200	−0.9704	0.341	−2.843	0.0509
	**700–2700**	**−2.1047**	**0.446**	**−4.716**	**< 0.0001**
	1200–1700	0.4394	0.353	1.244	0.8149
	1200–2200	−0.3185	0.386	−0.826	0.9629
	**1200–2700**	**−1.4528**	**0.481**	**−3.02**	**0.0304**
	1700–2200	−0.7578	0.309	−2.451	0.1391
	**1700–2700**	**−1.8922**	**0.422**	**−4.481**	**0.0001**
	2200–2700	−1.1343	0.45	−2.521	0.1178
Colour: Red	200–700	0.9948	0.491	2.026	0.3276
	200–1200	0.7372	0.517	1.426	0.7112
	200–1700	0.0291	0.425	0.068	1
	200–2200	0.1723	0.447	0.386	0.9989
	200–2700	0.3619	0.467	0.776	0.9717
	700–1200	−0.2576	0.476	−0.541	0.9945
	700–1700	−0.9657	0.374	−2.585	0.101
	700–2200	−0.8225	0.398	−2.064	0.3063
	700–2700	−0.6329	0.421	−1.504	0.6619
	1200–1700	−0.7081	0.407	−1.74	0.5053
	1200–2200	−0.5649	0.43	−1.314	0.7775
	1200–2700	−0.3752	0.451	−0.833	0.9615
	1700–2200	0.1432	0.313	0.458	0.9975
	1700–2700	0.3329	0.341	0.977	0.9255
	2200–2700	0.1897	0.368	0.516	0.9956
Colour: Orange	200–700	−0.5055	0.613	−0.824	0.9631
	200–1200	−0.4432	0.651	−0.681	0.9841
	200–1700	−0.2566	0.594	−0.432	0.9981
	200–2200	−0.1898	0.621	−0.306	0.9996
	200–2700	1.5404	0.896	1.719	0.519
	700–1200	0.0623	0.475	0.131	1
	700–1700	0.2489	0.394	0.632	0.9887
	700–2200	0.3158	0.433	0.73	0.9783
	700–2700	2.046	0.778	2.631	0.0899
	1200–1700	0.1866	0.45	0.414	0.9984
	1200–2200	0.2534	0.485	0.523	0.9953
	1200–2700	1.9837	0.808	2.455	0.1376
	1700–2200	0.0669	0.406	0.165	1
	1700–2700	1.7971	0.763	2.355	0.1722
	2200–2700	1.7302	0.784	2.208	0.234
Colour: Yellow	200–700	19.14	3926.922	0.005	1
	200–1200	1.176	1.247	0.943	0.9354
	200–1700	−0.554	0.792	−0.699	0.9821
	200–2200	0.977	1.023	0.954	0.9322
	200–2700	19.048	3919.617	0.005	1
	700–1200	−17.964	3926.922	−0.005	1
	700–1700	−19.694	3926.922	−0.005	1
	700–2200	−18.163	3926.922	−0.005	1
	700–2700	−0.092	5548.343	0	1
	1200–1700	−1.73	1.056	−1.639	0.5728
	1200–2200	−0.199	1.239	−0.161	1
	1200–2700	17.872	3919.617	0.005	1
	1700–2200	1.53	0.779	1.965	0.3624
	1700–2700	19.602	3919.617	0.005	1
	2200–2700	18.071	3919.617	0.005	1
Colour: Green	200–700	−1.7381	0.777	−2.236	0.2211
	200–1200	−0.5224	0.87	−0.6	0.9911
	200–1700	0.0339	0.829	0.041	1
	200–2200	0.0205	0.865	0.024	1
	200–2700	−0.3884	0.847	−0.458	0.9975
	700–1200	1.2157	0.541	2.246	0.2166
	**700–1700**	**1.772**	**0.471**	**3.761**	**0.0023**
	**700–2200**	**1.7585**	**0.532**	**3.305**	**0.0122**
	700–2700	1.3497	0.503	2.682	0.0788
	1200–1700	0.5563	0.613	0.908	0.9447
	1200–2200	0.5429	0.661	0.821	0.9637
	1200–2700	0.134	0.638	0.21	0.9999
	1700–2200	−0.0134	0.605	−0.022	1
	1700–2700	−0.4223	0.58	−0.728	0.9785
	2200–2700	−0.4088	0.63	−0.649	0.9872
Colour: Blue	200–700	0.554	16,245	0	1
	200–1200	−20.1244	12,246	−0.002	1
	200–1700	0.9521	16,048	0	1
	200–2200	0.6282	16,133	0	1
	200–2700	0.4619	16,232	0	1
	700–1200	−20.6784	10,674	−0.002	1
	700–1700	0.3981	14,883	0	1
	700–2200	0.0743	14,975	0	1
	700–2700	−0.092	15,082	0	1
	1200–1700	21.0765	10,371	0.002	1
	1200–2200	20.7527	10,502	0.002	1
	1200–2700	20.5864	10,655	0.002	1
	1700–2200	−0.3238	14,760	0	1
	1700–2700	−0.4901	14,869	0	1
	2200–2700	−0.1663	14,961	0	1
Colour: Purple	200–700	−16.139	1657.352	−0.01	1
	200–1200	−17.314	1657.352	−0.01	1
	200–1700	−15.253	1657.352	−0.009	1
	200–2200	−16.329	1657.352	−0.01	1
	200–2700	−18.449	1657.352	−0.011	1
	700–1200	−1.175	0.734	−1.601	0.5978
	700–1700	0.886	0.926	0.957	0.9312
	700–2200	−0.19	0.782	−0.243	0.9999
	**700–2700**	**−2.31**	**0.648**	**−3.563**	**0.0049**
	1200–1700	2.061	0.836	2.465	0.1347
	1200–2200	0.985	0.674	1.463	0.6882
	1200–2700	−1.135	0.512	−2.214	0.2309
	1700–2200	−1.076	0.879	−1.224	0.825
	**1700–2700**	**−3.196**	**0.762**	**−4.192**	**0.0004**
	**2200–2700**	**−2.12**	**0.579**	**−3.66**	**0.0034**
Colour: Pink	200–700	0.554	9853.381	0	1
	200–1200	−19.552	7427.736	−0.003	1
	200–1700	−18.253	7427.736	−0.002	1
	200–2200	−20.725	7427.736	−0.003	1
	200–2700	0.462	9845.471	0	1
	700–1200	−20.106	6474.4	−0.003	1
	700–1700	−18.807	6474.4	−0.003	1
	700–2200	−21.279	6474.4	−0.003	1
	700–2700	−0.092	9147.671	0	1
	1200–1700	1.299	0.93	1.397	0.7288
	1200–2200	−1.173	0.665	−1.763	0.4898
	1200–2700	20.014	6462.356	0.003	1
	**1700–2200**	**−2.472**	**0.772**	**−3.202**	**0.0171**
	1700–2700	18.715	6462.356	0.003	1
	2200–2700	21.187	6462.356	0.003	1
Colour: Brown	200–700	0.1442	0.894	0.161	1
	200–1200	0.0339	0.943	0.036	1
	200–1700	−0.9535	0.777	−1.227	0.8237
	200–2200	0.0205	0.865	0.024	1
	200–2700	18.0477	2377.368	0.008	1
	700–1200	−0.1103	0.788	−0.14	1
	700–1700	−1.0977	0.578	−1.899	0.4029
	700–2200	−0.1238	0.692	−0.179	1
	700–2700	17.9035	2377.368	0.008	1
	1200–1700	−0.9874	0.652	−1.515	0.6547
	1200–2200	−0.0134	0.754	−0.018	1
	1200–2700	18.0138	2377.368	0.008	1
	1700–2200	0.974	0.532	1.831	0.4454
	1700–2700	19.0012	2377.368	0.008	1
	2200–2700	18.0273	2377.368	0.008	1
Colour: Black	200–700	−0.11	0.54	−0.204	1
	200–1200	0.519	0.63	0.823	0.9633
	200–1700	1.138	0.584	1.948	0.3729
	200–2200	0.904	0.603	1.499	0.665
	200–2700	−0.308	0.538	−0.572	0.9928
	700–1200	0.629	0.524	1.199	0.8377
	700–1700	1.248	0.468	2.665	0.0823
	700–2200	1.013	0.491	2.063	0.3068
	700–2700	−0.198	0.409	−0.484	0.9967
	1200–1700	0.619	0.57	1.086	0.8873
	1200–2200	0.385	0.589	0.653	0.9868
	1200–2700	−0.827	0.523	−1.581	0.6111
	1700–2200	−0.234	0.54	−0.434	0.9981
	**1700–2700**	**−1.446**	**0.466**	**−3.1**	**0.0238**
	2200–2700	−1.212	0.49	−2.475	0.1316
Colour: White	200–700	1.2114	0.797	1.52	0.6513
	200–1200	0.7985	0.802	0.996	0.9194
	200–1700	0.7985	0.663	1.205	0.8348
	200–2200	0.7851	0.707	1.11	0.8776
	200–2700	1.119	0.798	1.403	0.7256
	700–1200	−0.4128	0.839	−0.492	0.9965
	700–1700	−0.4128	0.707	−0.584	0.9921
	700–2200	−0.4263	0.749	−0.569	0.993
	700–2700	−0.0924	0.835	−0.111	1
	1200–1700	0	0.713	0	1
	1200–2200	−0.0134	0.754	−0.018	1
	1200–2700	0.3205	0.84	0.382	0.999
	1700–2200	−0.0134	0.605	−0.022	1
	1700–2700	0.3205	0.708	0.452	0.9976
	2200–2700	0.3339	0.75	0.445	0.9978
Presentation: Cauliflorous	200–700	0.055	0.472	0.116	1
	200–1200	1.7698	0.714	2.478	0.1306
	200–1700	0.9987	0.475	2.1	0.287
	200–2200	1.0899	0.517	2.106	0.2839
	200–2700	19.8174	2363.894	0.008	1
	700–1200	1.7148	0.651	2.633	0.0894
	700–1700	0.9437	0.374	2.522	0.1177
	700–2200	1.0349	0.426	2.428	0.1465
	700–2700	19.7624	2363.894	0.008	1
	1200–1700	−0.7711	0.653	−1.18	0.8466
	1200–2200	−0.6799	0.685	−0.993	0.9203
	1200–2700	18.0476	2363.894	0.008	1
	1700–2200	0.0912	0.43	0.212	0.9999
	1700–2700	18.8187	2363.894	0.008	1
	2200–2700	18.7275	2363.894	0.008	1
Presentation: Ramiflorous	**200–700**	**−2.1332**	**0.498**	**−4.287**	**0.0003**
	**200–1200**	**−3.0406**	**0.592**	**−5.139**	**< 0.0001**
	**200–1700**	**−2.3611**	**0.474**	**−4.982**	**< 0.0001**
	**200–2200**	**−2.3408**	**0.494**	**−4.739**	**< 0.0001**
	**200–2700**	**−3.6083**	**0.605**	**−5.967**	**< 0.0001**
	700–1200	−0.9075	0.482	−1.884	0.4118
	700–1700	−0.2279	0.326	−0.699	0.9822
	700–2200	−0.2076	0.355	−0.586	0.992
	**700–2700**	**−1.4752**	**0.497**	**−2.965**	**0.0358**
	1200–1700	0.6795	0.457	1.486	0.6731
	1200–2200	0.6998	0.478	1.465	0.687
	1200–2700	−0.5677	0.592	−0.96	0.9306
	1700–2200	0.0203	0.321	0.063	1
	1700–2700	−1.2472	0.474	−2.632	0.0897
	2200–2700	−1.2675	0.494	−2.567	0.1055

*Note:* Estimate and standard error values are displayed, together with z ratios and *p* values adjusted for multiple comparisons. Significant comparisons are displayed in bold.

**TABLE A3:** List of fruiting plants identified to species level and thus included in phylogenetic analyses. Numbering of species corresponds to the phylogeny as depicted in Figure 2.

Number	Species
1	*Piper macropiper*
2	*Piper celtidiforme*
3	*Piper interruptum*
4	*Piper recessum*
5	*Zygogynum haplopus*
6	*Zygogynum oligostigma*
7	*Magnolia tsiampacca*
8	*Popowia pisocarpa*
9	*Myristica fatua*
10	*Myristica subalulata*
11	*Myristica filipes*
12	*Gymnacranthera paniculata*
13	*Litsea collina*
14	*Kibara coriacea*
15	*Pandanus kaernbachii*
16	*Caryota rumphiana*
17	*Smilax nova guineensis*
18	*Tinospora cordifolia*
19	*Eurya tigang*
20	*Saurauia conferta*
21	*Ardisia imperialis*
22	*Ardisia lanceolata*
23	*Maesa haplobotrys*
24	*Embelia cotinoides*
25	*Myrsine involucrata*
26	*Myrsine womersleyi*
27	*Pittosporum sinuatum*
28	*Scaevola oppositifolia*
29	*Polyosma cunninghamii*
30	*Cyrtandra erectiloba*
31	*Cyclophyllum brevipes*
32	*Psychotria micrococca*
33	*Psychotria leptothyrsa*
34	*Psychotria multicostata*
35	*Psychotria murmurensis*
36	*Ichnocarpus frutescens*
37	*Tabernaemontana pandacaqui*
38	*Cayratia trifolia*
39	*Leea indica*
40	*Medinilla lorentziana*
41	*Medinilla crassinervia*
42	*Decaspermum forbesii*
43	*Decaspermum alpinum*
44	*Sterculia schumanniana*
45	*Allophylus cobbe*
46	*Cupaniopsis acuticarpa*
47	*Harpullia ramiflora*
48	*Harpullia longipetala*
49	*Melicope elleryana*
50	*Micromelum minutum*
51	*Wenzelia dolichophylla*
52	*Aglaia tomentosa*
53	*Archidendron aruense*
54	*Xanthophyllum papuanum*
55	*Zehneria mucronata*
56	*Ziziphus angustifolia*
57	*Leucosyke australis*
58	*Artocarpus lakoocha*
59	*Streblus glaber*
60	*Ficus arfakensis*
61	*Ficus trichocerasa*
62	*Ficus hahliana*
63	*Ficus saccata*
64	*Ficus trachypison*
65	*Ficus subulata*
66	*Ficus wassa*
67	*Ficus badiopurpurea*
68	*Ficus microdictya*
69	*Ficus pungens*
70	*Ficus congesta*
71	*Ficus idiotricha*
72	*Ficus bernaysii*
73	*Ficus morobensis*
74	*Prunus oligantha*
75	*Prunus dolichobotrys*
76	*Breynia cernua*
77	*Glochidion angulatum*
78	*Bhesa archiboldiana*
79	*Casearia clutiifolia*
80	*Garcinia maluensis*
81	*Tripetalum cymosum*
82	*Homalanthus novoguineensis*
83	*Annesijoa novoguineensis*

**TABLE A4:** Results of phylogenetic null model analysis on individual fruit colours (top) and presentation types (bottom).

	*N*	Observed MPD	Randomised MPD mean	Randomised MPD SD	Standardised effect size	*p*
**Colour**						
Red	16	241.5	239.73	9.28	0.19	0.54
Orange	17	243.05	239.46	8.97	0.40	0.62
Yellow	5	242.55	239.27	21.57	0.15	0.48
Green	9	232.21	240.18	13.85	−0.58	0.23
Blue	1	NA	NA	NA	NA	NA
Purple	5	210.87	239.26	20.84	−1.36	0.07
Pink	3	228.09	236.6	36.73	−0.23	0.25
**Brown**	**12**	**213.91**	**239.16**	**11.43**	**−2.21**	**0.03**
Black	11	259.85	238.72	13.1	1.61	1
White	4	209.53	240.1	24.33	−1.26	0.11
**Presentation**						
**Cauliflorous**	**13**	**161.48**	**238.92**	**10.88**	**−7.12**	**< 0.01**
Ramiflorous	63	245.65	239.50	2.52	2.44	1

*Note:* Standardised effect sizes (SES) were obtained for each colour and presentation type by comparing the observed mean phylogenetic distance (MPD) between species displaying the trait with a null value obtained by randomising the tips of the phylogeny 999 times. Negative SES values indicate phylogenetic clustering, while positive values indicate phylogenetic evenness. Significant results (*p* < 0.05) are displayed in bold.
